# System Setup to Deliver Air Impact Forces to a Sheep Limb: Preparation for Model Development of Blast-Related Heterotopic Ossification

**DOI:** 10.2196/12107

**Published:** 2019-02-22

**Authors:** Dustin L Williams, Richard T Epperson, Nicholas B Taylor, Mattias B Nielsen, Brooke S Kawaguchi, David L Rothberg, Paul F Pasquina, Brad M Isaacson

**Affiliations:** 1 Bone & Joint Research Laboratory Department of Veterans Affairs Salt Lake City, UT United States; 2 Department of Orthopaedics University of Utah Salt Lake City, UT United States; 3 Department of Pathology University of Utah Salt Lake City, UT United States; 4 Department of Bioengineering University of Utah Salt Lake City, UT United States; 5 The Center for Rehabilitation Sciences Research, Department of Physical Medicine & Rehabilitation Uniformed Services University of the Health Sciences Bethesda, MD United States; 6 Department of Rehabilitation Walter Reed National Military Medical Center Bethesda, MD United States; 7 The Geneva Foundation Tacoma, WA United States

**Keywords:** blast, air, sheep, limb, heterotopic ossification

## Abstract

**Background:**

Heterotopic ossification (HO) is a significant complication for wounded warriors with traumatic limb loss. Although this pathologic condition negatively impacts the general population, ectopic bone has been observed with higher frequency for service members injured in Iraq and Afghanistan due to blast injuries. Several factors, including a traumatic insult, bioburden, tourniquet and wound vacuum usage, and bone fractures or fragments have been associated with increased HO for service members. A large combat-relevant animal model is needed to further understand ectopic bone etiology and develop new pragmatic solutions for reducing HO formation and recurrence.

**Objective:**

This study outlines the optimization of a blast system that may be used to simulate combat-relevant trauma for HO and replicate percussion blast experienced in theater.

**Methods:**

We tested the repeatability and reproducibility of an air impact device (AID) at various pressure settings and compared it with a model of blunt force trauma for HO induction. Furthermore, we assessed the ability of the higher-power air delivery system to injure host tissue, displace metal particulate, and disperse bone chips in cadaveric sheep limbs.

**Results:**

Data demonstrated that the air delivery setup generated battlefield-relevant blast forces. When the AID was charged to 40, 80, and 100 psi, the outputs were 229 (SD 13) N, 778 (SD 50) N, and 1085 (SD 114) N, respectively, compared with the blunt force model which proposed only 168 (SD 11) N. For the 100-psi AID setup, the force equaled a 5.8-kg charge weight of trinitrotoluene at a standoff distance of approximately 2.62 m, which would replicate a dismounted improvised explosive device blast in theater. Dispersion data showed that the delivery system would have the ability to cause host tissue trauma and effectively disperse metal particulate and host bone chips in local musculature compared with the standard blunt force model (13 mm vs 2 mm).

**Conclusions:**

Our data showed that a high-pressure AID was repeatable or reproducible, had the ability to function as a simulated battlefield blast that can model military HO scenarios, and will allow for factors including blast trauma to translate toward a large animal model.

## Introduction

Heterotopic ossification (HO) refers to ectopic bone formation, typically in residual limbs or periarticular regions following trauma, surgery, or injury [1]. This pathological process manifests outside the skeleton [2] and comprises a hybrid of cortical and cancellous bone [3]. HO is induced by damage to soft tissue and inflammation [4,5] and has been most frequently observed after combat-related trauma to service members with blast injuries [6].

Reviews of orthopedic injuries from Operation Iraqi Freedom and Operation Enduring Freedom have reported that approximately 70% of war wounds involved the musculoskeletal system [7], largely in part from the use of improvised explosive devices (IEDs) and rocket-propelled grenades. Given the intense nature of blast injuries, which require rapid tourniquet use, debridement, and surgical intervention, HO has been reported to occur in approximately 63%-65% of wounded service members with limb loss or major extremity injuries [8-10]. Reports of recent Operation Iraqi Freedom and Operation Enduring Freedom combat-related amputees with known HO have indicated that approximately 20%-40% of affected patients required surgery to excise their bony masses [10-13]. Symptomatic HO may delay rehabilitation regimens, as ectopic bone resection often requires modifications to prosthetic limb componentry and socket size [11,14].

The causative factors of HO development, especially in the case of blast injuries, are not well known. However, it has been hypothesized that contributing factors may include the following: (1) the blast, which generates extensive trauma and potential concomitant brain injury [15]; (2) tourniquet use, which alters local pH and creates a hypoxic environment [16]; (3) the presence of bacteria and biofilms [17]; (4) negative pressure wound therapy that may be used postinjury [9]; and (5) fractured bone, which may be dispersed into the musculature (clinical observations, unpublished data). To identify the various contributing factors for ectopic bone and to provide new evidence-based medicine that may inform clinical guidelines, animal models are currently being developed. However, as noted by Forsberg et al in *Burned to the Bone*, “one of the challenges preventing advances in this field has been the lack of robust animal models for HO” [18].

While rats and rabbits are the most commonly used animals for HO research, their bone growth rates are 600% and 40% higher, respectively, than those of humans [19,20]. This may limit the translatability of this work because HO has been documented to be more metabolically active than nonpathological osseous tissue [1,3,21-24]. Small animal models also cannot accurately reflect combat casualty care because variables such as serial debridement and negative pressure wound therapy must be omitted [25]. The most practical model, and one that is highly understudied, is the ovine model, which has almost identical mineral apposition rates [26] and bone ingrowth into intramedullary implants [27] compared with that of humans. Despite this evidence, only a single study by Walton et al [28] has evaluated HO development in an ovine model; the study results indicated that ectopic bone occurred only 17% of the time. However, Walton et al used blunt force rather than blast trauma, which does not replicate combat conditions, and histological data confirmed that HO formation did not occur. In an effort to address these limitations, preparations are underway to expand HO data collection into a large animal model that includes use of a simulated blast scenario. The first step in this process was to develop a system that could deliver a repeatable and reproducible high-pressure blast. This study outlines the optimization of a simulated blast system that may be translated to a large animal ovine model to assess the development of HO in blast-related scenarios.

## Methods

### Incident Pressures and Air Impact Device Selection

IEDs are often fabricated from 120-mm artillery rounds and contain approximately 5.8 kg trinitrotoluene (TNT) or its equivalent [29,30]. At a standoff distance of 5.5 m (one of the most commonly used measures for blast assessment), this yields an incident pressure of 110.9 kilopascal (kPa) based on the Kingery-Bulmash blast parameter calculator, which was used for calculating estimated incident pressures in this study [31]. Previous military blast injury models in rodents have utilized pressurized gas systems to mimic IED repercussions [29]. It has been shown that these system types result in incident pressures and other blast parameters, including waveform shape and impulse to detonation, that correlate with IED or other blast outputs [29,32].

In order to more closely simulate an IED or rocket-propelled grenade blast that may occur in theater, and to appropriately translate this to a large animal model, we consulted a special effects pyrotechnics expert who was familiar with the creation of controllable blasts using pressurized gas or air. We identified Martin Tornado Air Cannon with a 4-inch valve (Model BB4-12-28, Martin Engineering, Neponset, IL) as a viable option for simulating a blast. Based on technical sheets, the Tornado system provides rapid depressurization of air within 0.1 seconds [33]. Incident pressures were estimated to range between approximately 174 and 588 kPa (ie, 40-100 psi), consistent with what may be experienced in the range of a battlefield blast setting based on parameters from the Kingery-Bulmash blast parameter calculator [29-31]. The Martin Tornado Air Cannon and its setup, which have been termed the air impact device (AID), were assembled based on manufacturer’s recommendations and assessed initially for force output.

### Force Plate Testing

**Figure 1:**
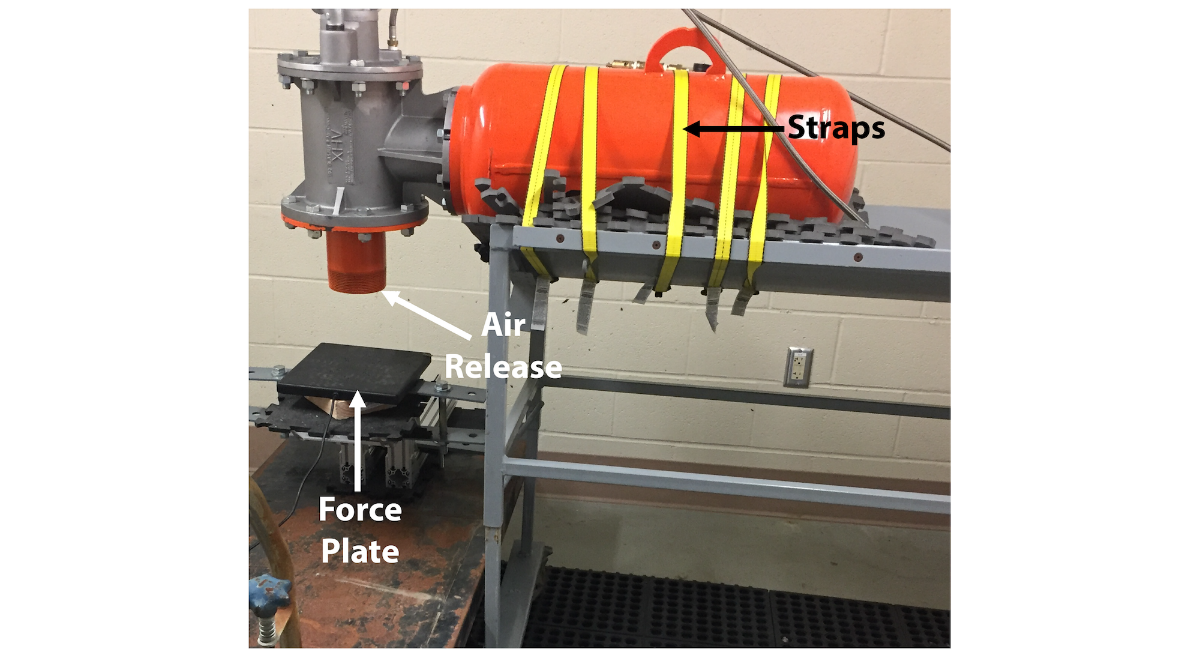
Setup of the air impact device for force plate analysis.

Animal limbs and carcasses for this and subsequent analyses were obtained from local butcher shops and from separate Institutional Animal Care and Use Committee-approved studies. To determine force outputs of the AID, NeuLog force plates (Amazon, Seattle, WA, Model Number NUL225) and accompanying sensors were purchased. The AID was secured to a metal cart using industrial strength tie-downs and situated such that the air release opening was directly over a force plate (Figure 1). Tie-down straps were used to secure the device. The NeuLog force plate was adjustable in height and tested at a distance of 2 inches from the AID air release opening to collect force plate data. The force plate was bolted to a custom-made aluminum stand, and tie-downs were used to secure the structure during AID discharge. The force plate was positioned 2 inches from the AID, and data was collected via universal serial bus to a general use Apple MacBook Pro on which NeuLog’s publicly available software had been downloaded. The AID was pressurized using a DeWalt fast charge air compressor and tested at pressures of 40, 80, and 100 psi. These settings were assessed experimentally to, in future, determine their ability to cause localized trauma but be within a factor of safety to not cause ovine fractures at this stage of the model. Once pressurized, the AID was discharged. Data were collected with 10 repeat measurements at each psi.

In order to establish baseline force outputs for ovine-induced trauma, we also reproduced the method performed by Walton et al [28], which required a weight of approximately 3.5 kg (head of a sledge hammer) to be dropped from a height of 1 meter. The force of this impact was recorded 10 times for comparison against the AID outcomes. Once force data were collected, testing of the AID system was advanced to *ex vivo* cadaveric sheep limb analysis.

### Cadaveric Limb Testing

**Figure 2:**
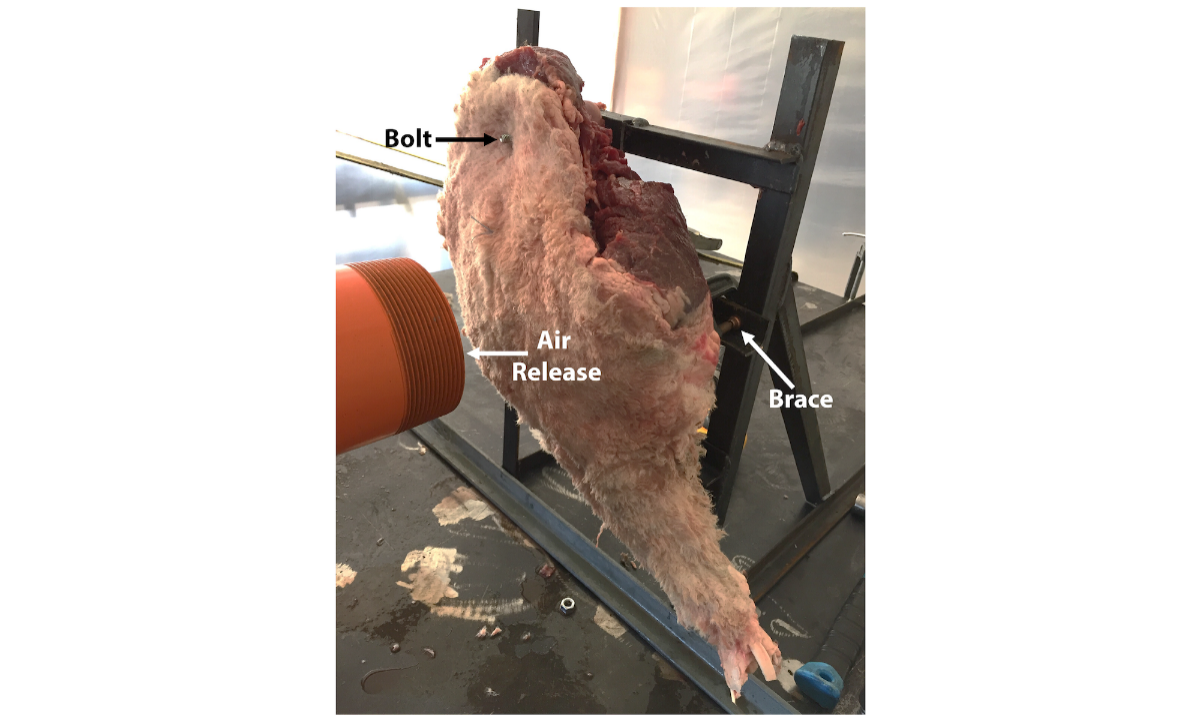
Representative image of a cadaveric sheep limb attached to a metal support frame for air impact device (AID) testing.

**Figure 3:**
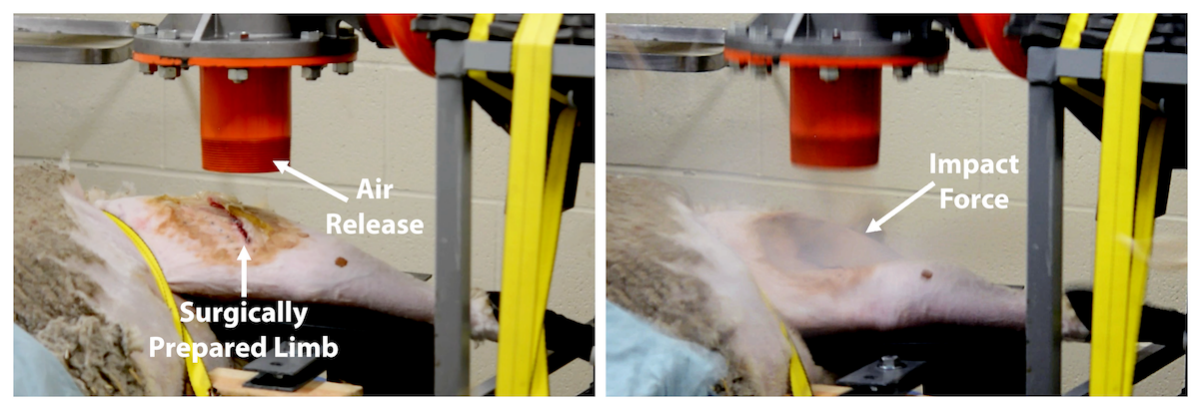
Impact of the air impact device (AID) blast on a cadaveric sheep. Left: Representative image showing a surgically prepared limb prior to an AID blast. The air release opening of the AID was positioned directly above the incision; Right: Still shot showing the effect of the impact force of the AID blast.

The AID blast was evaluated on 8 ovine carcass limbs and 4 whole sheep cadavers to characterize the effect of the air blast, to ensure that the force would not generate a localized fracture, and to optimize the surgical model. To ensure that the limb was in the same position between each blast, a support frame was custom welded and a brace secured in the midregion of the limb to prevent flexion and fracture. This was done for disarticulated limbs (Figure 2), as well as whole carcasses (Figure 3). The AID was placed 2 inches from the cadaveric limb (Figure 2) to be consistent with the force plate testing. Bolts were used to attach the limb to the metal frame. Note the metal brace on the back of the limb provided support to the femur and prevented breakage or severe ligament damage from occurring (Figure 2). Radiographs were taken following AID blasts to verify postprocedure bone integrity.

### Mock Shrapnel Displacement Testing

**Figure 4:**
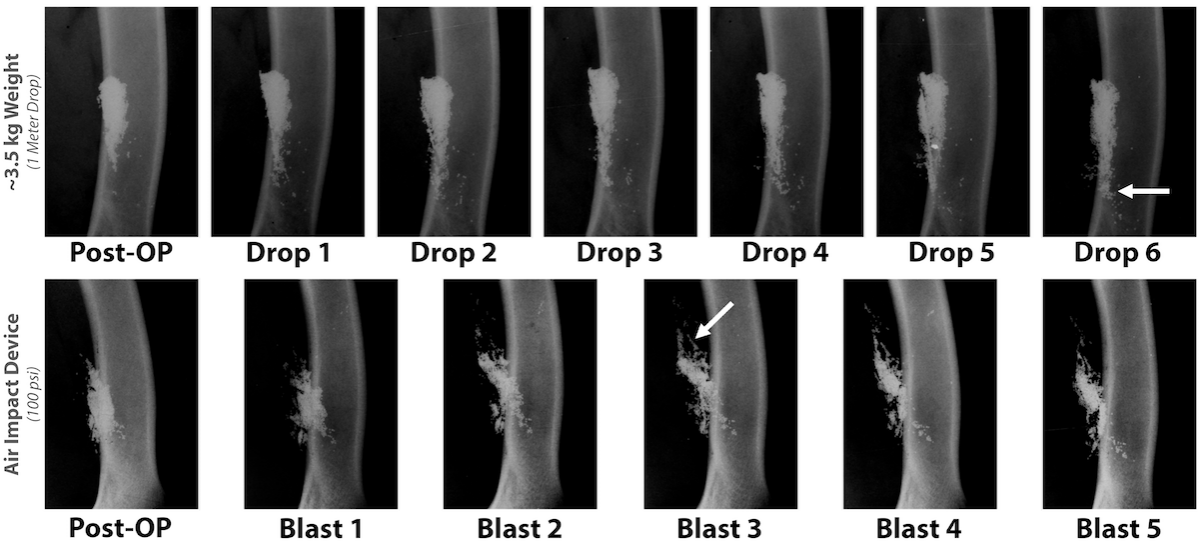
Radiograph demonstrating that following air impact device exposure or blunt force trauma, femora were intact and not fractured. In addition, images show dispersion of Cobalt-Chromium beads (white).

IED blast injuries often afflict wounded warriors with shrapnel in the distal limbs. To model this scenario and assess the ability of the AID blast procedure to disperse simulated shrapnel particles into the musculature of cadaveric sheep limbs, a whole carcass was obtained. An incision was made in the midshaft region of a femur. Deep tissue was dissected longitudinally until bone was exposed. A 2.5-g mixture of Cobalt-Chromium (CoCr) beads having a diameter of approximately 0.5 mm was suspended in 5 mL saline solution. The slurry was pipetted over the bone surface. A radiograph was obtained to determine the initial distribution of the CoCr beads (Figure 4).

Once placement was determined, the midshaft incision was sutured closed and covered with clear adhesive (ie, Tegaderm), and the AID discharge procedure, as outlined above, was performed. To assess for particulate dispersion, the blasting procedure was repeated 5 times. After each blast, a radiograph was obtained to track the displacement of CoCr beads in the deep tissue. This process was repeated in 2 cadaveric limbs.

For comparison, CoCr displacement testing was also performed using the Walton et al [28] method. More specifically, a sheep cadaver was obtained, an incision made in the midshaft region of the femur as described, and a 3.5-kg weight was dropped from 1-m height. Bead placement was again imaged using radiography at time zero and after each drop of the weight to track the movement of the CoCr beads.

**Figure 5:**
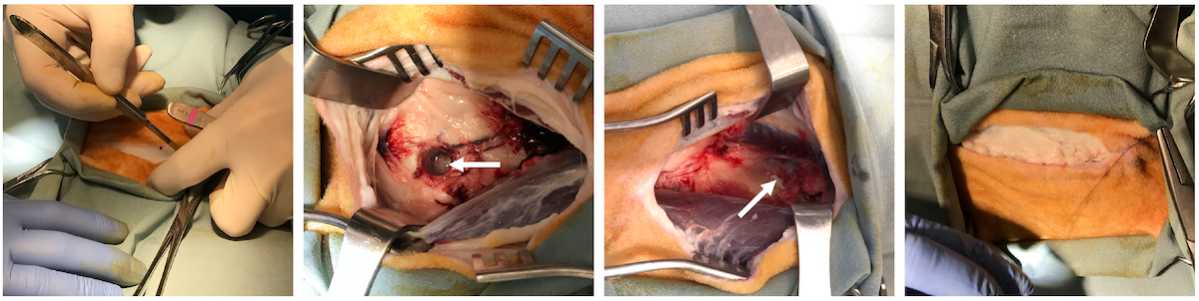
Photography demonstrating a mock surgery on a cadaver sheep for bone chip collection and placement. Left to right: Incision being made toward the distal end of the femur. A 10-mm bone core (arrow) was taken from the distal femur. Bone chips (arrow) were placed on the exposed midshaft of the femur. The incision site was sutured closed. Note that the fascia was also closed by suturing to ensure that the air impact device would not result in surgical site dehiscence.

**Figure 6:**
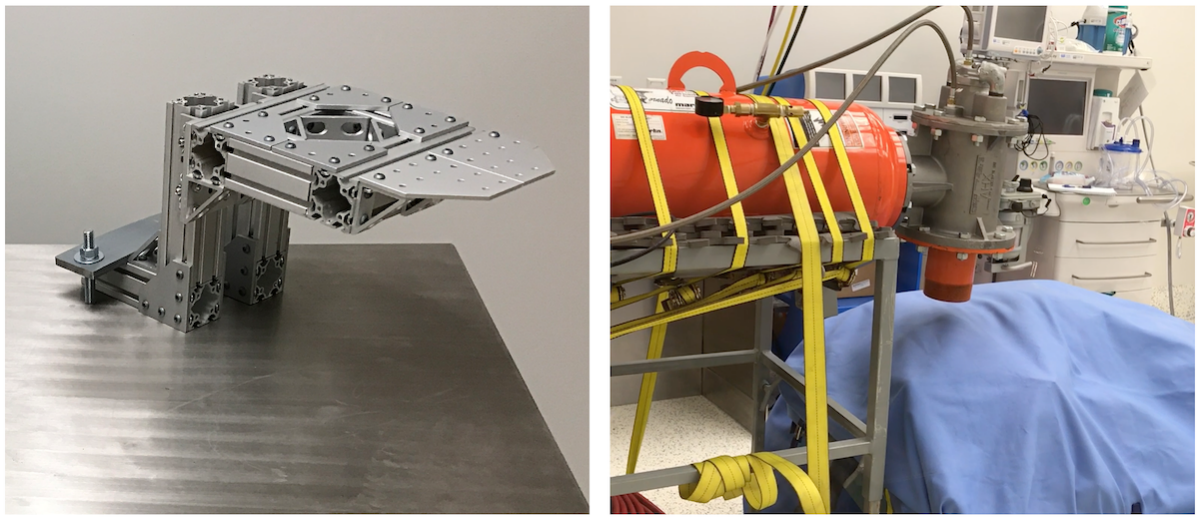
Photography demonstrating the AID blast. Left: A custom limb support created from 80/20 aluminum. This ensured the femur was supported during the lateral air impact device (AID) blast. Right: The final setup of the AID blast over a surgically operated leg.

**Figure 7:**
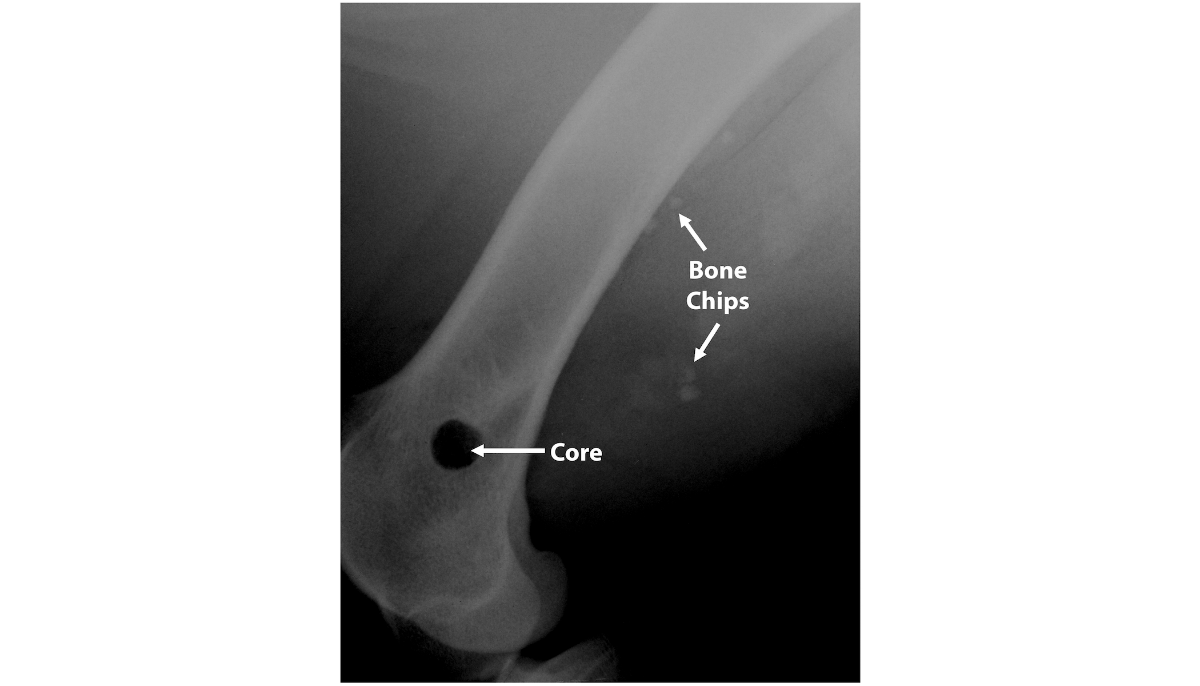
Radiograph obtained after 5 AID blasts revealed that the bone chips placed on the lateral side of the femur had migrated posteriorly as well as into the adjacent muscle tissue.

In addition to assessing the displacement of CoCr beads, testing was also performed to determine whether the AID could cause host bone chips or fragments to disperse in cadaveric sheep tissue. A mock surgery was performed wherein a bone core of approximately 10 mm was taken from the distal femur. Bone chips were created using a rongeur, mixed with saline to create a slurry, and placed in apposition to the bone in the midshaft of the femur (Figure 5). The sheep was covered with a drape to prevent any contamination that may have been forced though the incision site and to protect equipment in the room (Figure 6). Radiography was obtained after AID blasts (Figure 7).

## Results

### Force Plate Testing

Results from the force plate portion of testing showed that the air discharge forces of the AID exceeded the force achieved by Walton et al, which required dropping a 3.5-kg weight from a 1-m height (Figure 3). When the AID was pressurized to 40 psi, the air volume was 6.2 cubic feet [33]. At this psi, the incident pressure was 174 kPa, which would equate to a 5.8-kg charge weight of TNT at a standoff distance of approximately 4.5 m [31]. Incident pressure in this case was defined as a free air burst, meaning a burst that had no contact with the ground before striking an object [34]. The force output was 229 (SD 13) N. At 80 psi, the air volume was 10.7 cubic feet [33]. At this psi, the incident pressure was 450 kPa, which would equate approximately to a 5.8-kg charge weight of TNT at a standoff distance of approximately 2.95 m [31]. The force output was 778 (SD 50) N. At 100 psi, the air volume was 12.9 cubic feet [33]. At this psi, the incident pressure was 588 kPa, which would equate approximately to a 5.8-kg charge weight of TNT at a standoff distance of approximately 2.62 m [31]. The force output was 1085 (SD 114) N. Testing did not go higher than 100 psi given that the AID began to have connection leaks at higher pressures.

The force of dropping the 3.5-kg weight was 168 (SD 11) N. Taken together, the data indicated that the AID resulted in a force output that was approximately 7× greater than the dropped weight (Tables 1 and 2) and provided incident pressures that may more closely model an IED.

### Cadaveric Limb Testing

Tests from the cadaveric limbs indicated that with a support bar in place (Figure 2), limbs did not fracture. However, it was found that when an incision was present in the leg, the rapid discharge of air opened the incision and created a pocket that compromised the subdermal tissues. To mitigate this outcome, the incision site was covered with durable plastic, such as Tegaderm, which prevented the explosive air from entering the incision site and compromising the musculature. Whole carcass testing was performed in a horizontal plane to more closely simulate a sheep that would be lying on a table for a procedure to be performed.

**Table 1:** Force plate data output comparisons.

Group	Force output (N)		
	Mean (SD)	95% CI	Minimum, maximum
~3.5-kg weight	168 (11)	159-177	148, 179
AID^a^ (40 psi)	229 (13)	217-241	214, 245
AID (80 psi)	778 (50)	745-811	732, 881
AID (1000 psi)	1080 (114)	968-1190	1008, 1252

^a^AID: air impact device.

**Table 2:** Force plate data statistical comparisons.

Group	*P* value^a^
~3.5-kg weight versus AID^b^ (40 psi)	<.001
~3.5-kg weight versus AID (80 psi)	<.001
~3.5-kg weight versus AID (100 psi)	<.001
AID (40 psi) versus AID (80 psi)	<.001
AID (40 psi) versus AID (100 psi)	<.001
AID (80 psi) versus AID (100 psi)	<.001

^a^*P*<.05 is significant.

^b^AID: air impact device.

### Mock Shrapnel and Bone Displacement Testing

Results from the mock shrapnel displacement testing showed that the AID discharge procedure dispersed CoCr beads within the musculature of a cadaveric sheep limb (Figure 4). More specifically, groups of beads were tracked and dispersed to a distance of approximately 2.7 mm with each blast that was performed. By the fifth blast, beads resided approximately 13.3 mm distal to their start point. The data also indicated that the sheep limbs were able to withstand multiple sequential blasts. More specifically, radiographs indicated that the limbs did not fracture following multiple AID discharges (Figure 4).

For comparison, the process of dropping a 3.5-kg weight on the limb resulted in minimal movement of the CoCr beads with each sequential hit (Figure 4). Beads primarily tracked parallel to the bone and may have been an artifact from motion during the capturing of the radiographs or as saline drained through the surgical pocket that was created (Figure 4). By the fifth drop of the weight, beads had dispersed by approximately 2 mm or less into the surrounding tissue regions.

## Discussion

### Principal Findings

The setup of an AID system described herein generated repeatable and reproducible blast of pressurized air that resulted in a force of approximately 1100 N. This may cause significant trauma to local tissue without compromising the underlying skeletal structure of a large animal (which may be critically important for a translatable animal model because lameness and extreme discomfort may necessitate euthanasia). The forces generated in our model were approximately 7× greater than those generated in the blunt force trauma model previously developed to induce ectopic bone [28].

The delivery of a pressurized blast of air was consistent with previous animal studies and incident pressures that may be present in an IED blast [29]. However, the overall goal was not to create massive polytrauma, but rather consistent blasts of air. The AID used in this model also demonstrated that it could effectively disperse metal particulate within the muscle, which would be expected with percussion blasts. Metal beads tracked parallel to the bone following the weight drop, displacing within the soft tissue planes of our intermuscular approach. In contrast, beads that dispersed into the musculature following AID blasts appeared to disperse in a radial pattern created by the pressurized blast of air.

Bone chips or fragments were also found to be affected by the AID. This may be particularly important when the animal model portion of testing begins because in a battlefield-relevant scenario, bone chips or fragments are a common result of blast-related trauma. These data indicate that as this work progresses toward animal modeling, clinically relevant outcomes may be achieved. Current testing has been limited to *ex vivo* analysis. Live animal modeling will be needed to determine whether these data model parameters are safe and effective. *In vivo* data will also reveal whether an approach of highly pressurized air, as opposed to blunt force, will lead to higher rates of HO formation in an ovine model.

### Conclusion

HO negatively affects the quality of life for service members and those in the public sector. For example, the pathology can inhibit the ability of those with limb loss to effectively use prosthetic socket systems due to pain as soft tissues compress against bony HO. This in turn delays rehabilitation and, in some cases, requires surgical excision. Methods to better understand the etiology and ectopic bone mitigation will improve clinical outcomes. This study outlines the setup of a high-pressure air blast system to simulate combat-related trauma that may lead to future HO manifestation.

## Abbreviations

AID: air impact device
HO: heterotopic ossification
IED: improvised explosive device
TNT: trinitrotoluene
